# Notch and VEGF pathways play distinct but complementary roles in tumor angiogenesis

**DOI:** 10.1186/2045-824X-5-17

**Published:** 2013-09-25

**Authors:** Sonia L Hernandez, Debarshi Banerjee, Alejandro Garcia, Thaned Kangsamaksin, Wei-Yi Cheng, Dimitris Anastassiou, Yasuhiro Funahashi, Angela Kadenhe-Chiweshe, Carrie J Shawber, Jan K Kitajewski, Jessica J Kandel, Darrell J Yamashiro

**Affiliations:** 1 Department of Pediatrics, Columbia University Medical Center, New York, NY, USA; 2 Department of Surgery, Columbia University Medical Center, New York, NY, USA; 3 Department of Obstetrics and Gynecology, Columbia University Medical Center, New York, NY, USA; 4 Center for Computational Biology and Bioinformatics, Columbia University, New York, NY, USA; 5 Department of Pathology and Cell Biology, Columbia University Medical Center, New York, NY, USA

**Keywords:** Neuroblastoma, Vascular endothelial growth factor, Notch, Angiogenesis, Bevacizumab

## Abstract

**Background:**

Anti-angiogenesis is a validated strategy to treat cancer, with efficacy in controlling both primary tumor growth and metastasis. The role of the Notch family of proteins in tumor angiogenesis is still emerging, but recent data suggest that Notch signaling may function in the physiologic response to loss of VEGF signaling, and thus participate in tumor adaptation to VEGF inhibitors.

**Methods:**

We asked whether combining Notch and VEGF blockade would enhance suppression of tumor angiogenesis and growth, using the NGP neuroblastoma model. NGP tumors were engineered to express a Notch1 decoy construct, which restricts Notch signaling, and then treated with either the anti-VEGF antibody bevacizumab or vehicle.

**Results:**

Combining Notch and VEGF blockade led to blood vessel regression, increasing endothelial cell apoptosis and disrupting pericyte coverage of endothelial cells. Combined Notch and VEGF blockade did not affect tumor weight, but did additively reduce tumor viability.

**Conclusions:**

Our results indicate that Notch and VEGF pathways play distinct but complementary roles in tumor angiogenesis, and show that concurrent blockade disrupts primary tumor vasculature and viability further than inhibition of either pathway alone.

## Background

The anti-vascular endothelial growth factor (VEGF) antibody bevacizumab (BV) is clinically validated to treat human cancers in adults, and is currently being evaluated as a therapy for pediatric tumors, including neuroblastoma [1]. However, it appears that essentially all patients, even those who respond to BV initially, will ultimately progress despite ongoing treatment. Thus, a similar limitation is anticipated in children treated with this agent. The mechanisms of tumor progression during BV treatment are not well understood.

We have previously shown that VEGF inhibition disrupts the blood vessels of the neuroblastoma NGP xenograft model, but resistance occurs [2-4]. VEGF blockade can increase cooption of existing vascular structures by encroaching tumors [2], a mechanism which may partly compensate for defective VEGF signaling. An additional mechanism for tumor progression during VEGF blockade could be the activation of an alternative angiogenic pathway. In support of this concept, we have shown that the Notch pathway is up-regulated in neuroblastoma when the VEGF receptor 2 (VEGFR2) is blocked with the anti-VEGFR2 antibody DC101 [3].

To examine the role of Notch in tumor angiogenesis, we have used the Notch1 decoy (N1D), a soluble construct derived from the extracellular domain of the Notch1 receptor that blocks Notch activation by sequestering Notch ligands [5]. Notch blockade using the N1D disrupts tumor vasculature and decreases tumor viability [5]. Based on our previous studies, we thus hypothesized that blocking the Notch and VEGF pathways simultaneously would disrupt the vasculature and inhibit tumor growth more effectively than either agent alone. To test this hypothesis, we evaluated the effects of expressing N1D in combination with bevacizumab (BV) in the NGP neuroblastoma xenograft model. Here, we report that combining N1D with BV had a profound effect on the tumor viability and the vasculature of NGP xenografts. N1D and BV additively increased tumor endothelial cell apoptosis, leading to blood vessel regression, and cell death.

## Methods

### Transfections and cell culture

NGP-LacZ and NGP-N1D cells were maintained as described [5]. pLKO.1 empty vector (control) and pLKO.1 Notch1 shRNA (Notch1 KD) plasmids (Sigma Aldrich) were stably transfected into NGP cells, selected and maintained in 1 mg/ml puromycin (Sigma Aldrich).

### NGP xenograft model

1 × 10^6^ cells were injected intrarenally into 4–6 week old NCR female nude mice (Taconic, Germantown, NY) as previously described [3]. All animal experiments were approved by the Columbia University Institutional Animal Care and Use Committee. At sacrifice, organs were harvested and weighed as previously described [3].

### Bevacizumab and placebo administration

NGP-LacZ and NGP-Notch1 decoy (N1D) tumor-bearing mice were randomized one week after implantation. At this time, 28 mice in each group were treated with 100 μg mouse albumin (Sigma Aldrich) (placebo), and another 28 with 250 μg bevacizumab (10 mg/kg BV, Genentech) by intraperitoneal injection biweekly until time of sacrifice. Both the N1D and BV are fused to a human Fc fragment. Immunohistochemistry (Additional file 1: Figure S1A) and SDS-PAGE (Additional file 1: Figure S1B) to human Fc was performed on tumors, and confirmed expression of N1D in the tumors at time of sacrifice. Since BV is also fused to human Fc, it could also be detected in the NGP-LacZ + BV and NGP-N1D + BV tumors by SDS-PAGE (Additional file 1: Figure S1B, lower band).

### Reagents

Primary antibodies: αSMA (Lab vision), cleaved Notch1 (Cell Signaling), PECAM-1 (Angioproteomie), Notch1 (Upstate), collagen IV (Cosmobio), biotinylated hFc (Jackson Labs). Alexafluor probes (Invitrogen) were used for fluorescent stains.

### Hypoxyprobe and quantification

Thirty minutes prior to sacrifice, mice received an intraperitoneal injection of 0.1 ml of pimonidazole (Hypoxyprobe®, Chemicon), and paraffin sections were used to stain for pimonidazole with MAB-1 (Chemicon), following the manufacturer’s instructions. Four tumors per treatment group were quantified, with an average of 10 images taken from each tumor section at 20×. The images were then analyzed using Adobe Photoshop using an arbitrary threshold applied to all images.

### Lectin perfusion and quantification

An intracardiac injection of 100 μl of Fluorescein labeled *Lycopersicon esculentum* (Vector Labs) was delivered 2 minutes prior to 1% PFA and PBS perfusion, followed by tumor collection. Tumors were fixed in 4% PFA and 50 μm thick sections were analyzed using confocal microscopy.

### TUNEL assay quantification

TUNEL staining and quantification was performed as described [5]. An average of 20 images from each tumor section, and 5 tumors per group were analyzed.

### Collagen IV, PECAM-1 and EC apoptosis quantifications

Fresh-frozen, serial sections were used for Collagen IV, PECAM-1 and EC apoptosis quantifications. Viable tumor areas immunostained for collagen IV or PECAM-1 were photographed at 10× (Collagen IV) or 20× (PECAM-1). An average of 15 (Collagen IV) or 30 (PECAM-1) images per tumor, and three tumors per group were analyzed. For EC quantification, TUNEL, PECAM-1, stained sections were mounted with DAPI (Vector) and images obtained at 40× magnification.

### Cooption quantification

5 μm sections of paraffin-embedded livers or kidneys were stained with hematoxylin and eosin (H&E). Tumor sections were scanned using an Epson 4870 Scanner. Glomeruli surrounded by at least one layer of tumor cells were counted as coopted vasculature. One section per tumor, and 28–30 tumors per group were analyzed.

### VEGF ELISA

3 × 10^6^ NGP-LacZ or NGP-N1D cells were plated on a 10 cm dish, and cultured in 6 ml of serum-free EBM-2 (Invitrogen) for 48 hrs. Supernatant from three independent collections was measured, using a human VEGF ELISA kit (R&D) following the manufacturer’s instructions.

### HUVEC cell death and BrdU incorporation assays

8,000 HUVEC per well (48 well plate) were seeded on a fibrinogen gel (Sigma), in basal EBM-2 media (Lonza). Two hrs later, media was replaced by conditioned media (CM) from NGP-LacZ or NGP-N1D cells (3×10^6^ cells per 10 cm^2^ plate, cultured for 24 hrs in EBM-2 basal media. We added 50 ng/ml BV or 20 ng/ml rhVEGF165 (R&D) to the CM. For BrdU assays, 2 μl BrdU (Millipore) was added per ml of CM. Cell death assays (Roche) or BrdU incorporation assays (Millipore) were measured 23 hrs later, following the manufacturer’s instructions.

### Immunoprecipitation and Western Blots

Tumors were lysed in a ratio of 100 ml of lysis buffer to 10 mg of tumor. 100 μl of tumor lysate was immunoprecipitated with A/G agarose beads (Santa Cruz Biotechnology) and blotted with anti-human Fc (Pierce).

### Reverse transcription and quantitative PCR

RNA was extracted from cells following the manufacturer’s instructions (Qiagen). Tissue RNA extraction was performed with Ambion ToTALLY RNA kit (Ambion) following the manufacturer’s specifications. RNA from tissue or cells was reverse transcribed following the manufacturer’s instructions (Invitrogen, DNAseI and Superscript). Quantitative PCR was performed with the Taqman system (Applied Biosystems) following the manufacturer’s specifications, using primers designed not to amplify genomic DNA. GeNorm was used to select the normalization values out of 6 housekeeping genes.

### Microarrays and probes preparation

HG-U133A 2.0 (human genome) Gene Chips (Affymetrix, Santa Clara, CA) were used to investigate gene expression in xenograft tumors. The cRNA probes were synthesized as recommended by Affymetrix, purified using RNeasy and fragmented according to the Affymetrix protocol, and 15 μg of biotinylated cRNA were hybridized to the microarrays. The samples were scanned with Affymetrix Gene Chip Scanner 3000. The data set, corresponding to 24 tumors (6 arrays per group), has been deposited in NCBI’s Gene Expression Omnibus (GEO) and is available through accession number GSE (pending). Data were RMA normalized using the Bioconductor open source software.

#### Statistical analysis

Statistical analysis was performed using the Prism 5 software (GraphPad Software, Inc. La Jolla, CA). One-way analysis of variance was performed with post-hoc analysis by Tukey’s Multiple Comparison Test. Graphs represent mean and standard deviation in all cases.

## Results

### Combined blockade of notch and VEGF increases necrosis and apoptosis in primary tumors

**Figure 1:**
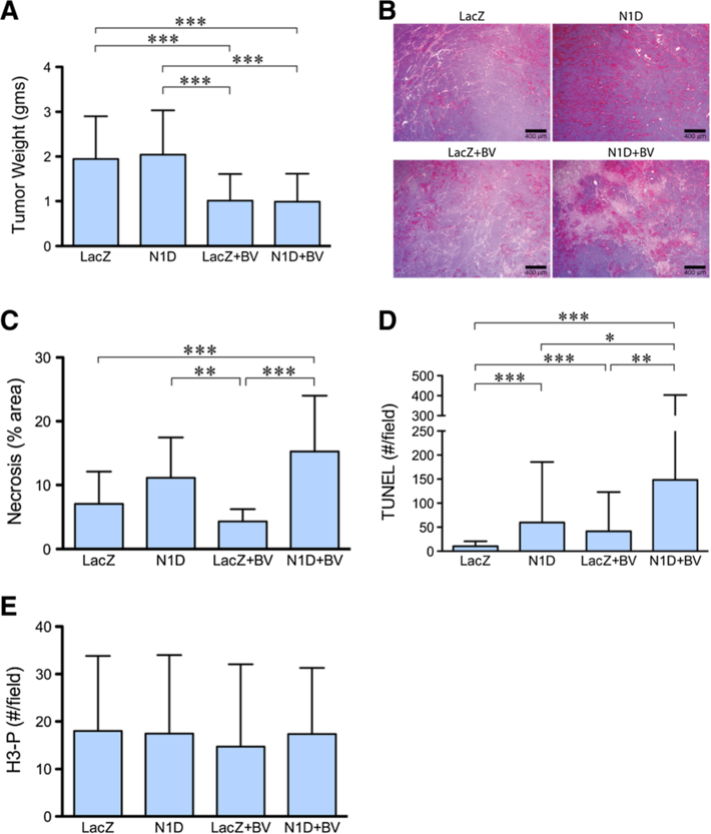
**Combined Notch and VEGF increases tumor necrosis and apoptosis. (A)** NGP-N1D tumors did not have different tumor weights than NGP-LacZ controls. BV treatment reduced tumor weights compared to controls and NGP-Notch1 decoy. Combining Notch1 decoy with BV reduced tumor weights compared to NGP-LacZ controls and NGP-N1D Error bars represent SD. ***P < 0.001. **(B)** NGP**-**N1D + BV tumors had increased necrosis as seen by H&E. Bar = 400 μm. **(C)** Quantification of necrosis showed NGP-N1D + BV with increased necrosis. Error bars represent SD. **P < 0.01, ***P < 0.001. **(D)** NGP-N1D and NGP-LacZ + BV tumors had increased numbers of TUNEL positive counts per field compared to NGP-LacZ controls. An additive increase was observed in NGP-N1D + BV tumors (p < 0.001). Error bars represent SD. *P < 0.05, **P < 0.01, ***P < 0.001. **(E)** Proliferation in tumors was assessed by phosphorylated-Histone H3 (H3-P) IHC. There was no significant difference in proliferation. Error bars represent SD. P = n.s.

We have previously shown that inhibiting Notch signaling with the soluble Notch1 decoy (N1D) decreased viability (but not tumor weight), disrupted vasculature, disorganized the interaction of endothelial cells with pericytes in neuroblastoma NGP xenografts [5]. Given the critical role of VEGF in tumor angiogenesis, we hypothesized that combining Notch inhibition with VEGF blockade would synergistically inhibit tumor growth and angiogenesis. NGP-N1D cells or control NGP-LacZ cells were implanted into the left kidney of athymic mice. The mice were then randomized to treatment with either placebo or BV, and sacrificed at 5.5 weeks. Consistent with our previous data [5], there was no difference in tumor weight between control NGP-LacZ and NGP-N1D (Figure 1A). VEGF blockade with BV, however, reduced NGP mean tumor weight by 61% (p < 0.001) compared to NGP-LacZ. Combined treatment with N1D and BV (NGP-N1D + BV), reduced mean tumor weight by a similar extent, 60%, as compared to NGP-LacZ, (p < 0.001).

Although there was no difference in tumor weight as compared with BV treatment alone, the NGP-N1D + BV tumors were noticeably more necrotic (Figure 1B), which was confirmed by quantification of necrosis in H&E sections (Figure 1C). Trichrome staining confirmed that these differences were due to necrosis and not fibrosis (data not shown). When assessed by TUNEL, the NGP-N1D + BV tumors exhibited significantly more apoptosis then NGP-LacZ controls (14.4 fold), BV alone (3.6 fold), or N1D alone (2.5 fold) (Figure 1D). Blockade of Notch or VEGF did not have a direct effect on tumor cell proliferation, as there was no difference in phospho-histone H3 positive cells in areas of viable tumor (Figure 1E). These data indicate that combined Notch and VEGF blockade additively decreases tumor viability.

### Combined blockade of notch and VEGF markedly decreases perfusion

**Figure 2:**
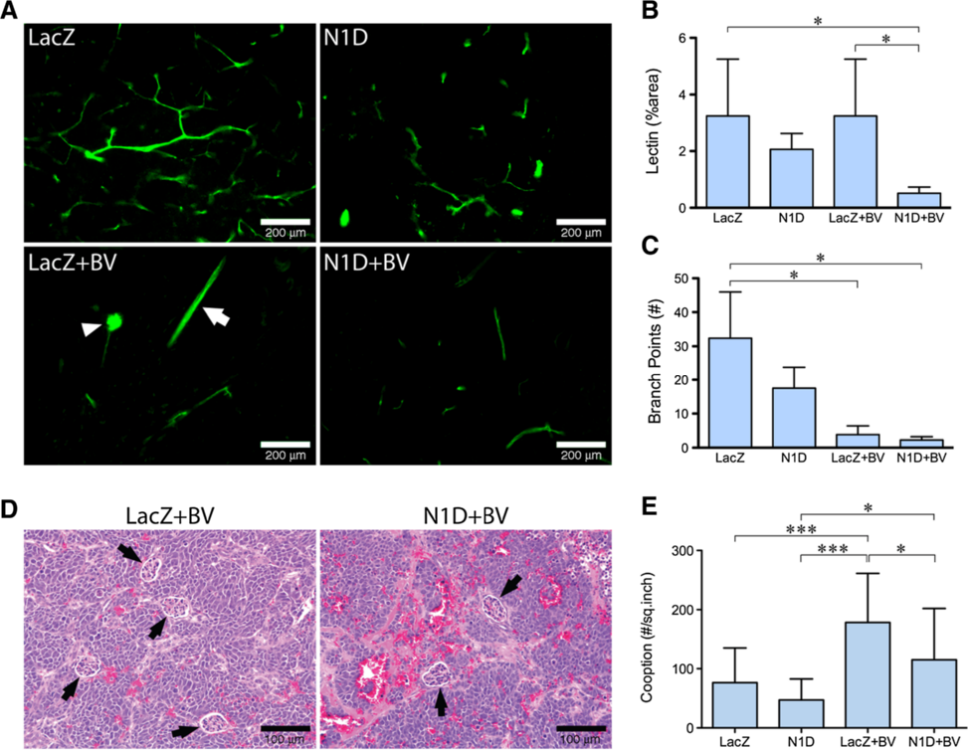
**Combined Notch and VEGF blockade decreases perfusion and branching. (A)** Lectin perfusion showed marked reduction in perfusion in NGP-N1D + BV. Bar = 200 μm. **(B)** Lectin perfusion showed marked reduction in perfusion in NGP-N1D + BV. Error bars represent SD. *P < 0.05.** (C)** Marked decrease in branches in NGP-LacZ + BV and NGP-N1D + BV. Error bars represent SD. *P < 0.05. **(D)** Representative H&E of NGP-LacZ + BV and NGP-N1D + BV tumors with coopted glomeruli (black arrowheads). Bar = 100 μm. **(E)** Compared to NGP-LacZ controls, NGP-LacZ + BV tumors had a higher number of coopted glomeruli per square inch. NGP-N1D tumors were not different from NGP-LacZ controls. NGP-N1D + BV tumors had less coopted glomeruli than NGP-LacZ + BV tumors. Error bars represent SD. *P < 0.05, ***P < 0.001.

We next evaluated the effect of Notch and VEGF blockade on lectin perfusion (Figure 2A,B). We found that with combined blockade (NGP-N1D + BV), there was a paucity of vessels, with a significantly reduced (P < 0.05) lectin-positive area (16% of control NGP-LacZ, Figure 2B). Blockade with N1D or BV alone did not reduce overall perfusion as measured by lectin-positive area (Figure 2B). However, in the case of BV, bound lectin outlined predominantly larger vessels (Figure 2A, arrow), or coopted renal glomeruli (Figure 2A, arrowhead), which contribute disproportionally to the total lectin-positive area. There was a marked reduction in vessel branching when VEGF was blocked (Figure 2C), either with BV alone (12%) or with N1D + BV (7%), indicating that VEGF blockade inhibits the small sprouts that initiate neoangiogenesis.

The decrease in lectin-positive area for NGP-N1D + BV relative to NGP-BV tumors suggested that there could be a decrease in the number of coopted glomeruli. We quantified coopted vessels by counting the number of glomeruli surrounded by tumor cells in H&E sections (Figure 2D, arrows). The number of coopted glomeruli in NGP-LacZ + BV tumors was increased 2.3 fold compared to NGP-LacZ tumors (Figure 2E). Notch blockade significantly attenuated this BV-induced increase in cooption, with NGP-N1D + BV tumors having significantly fewer coopted glomeruli as compared to the NGP-LacZ + BV group (115 vs 178, respectively, Figure 2E). These findings suggest that Notch blockade attenuates BV-induced cooption in NGP xenografts. Furthermore, immunostaining for cleaved Notch1, reflecting processing of the Notch receptor after ligand binding, showed increased activated Notch1 in coopted glomeruli as a result of BV treatment in NGP tumors (Additional file 2: Figure S2). These data implicate Notch signaling in BV-induced cooption of host vasculature.

### Blockade of VEGF results in tumor hypoxia

**Figure 3:**
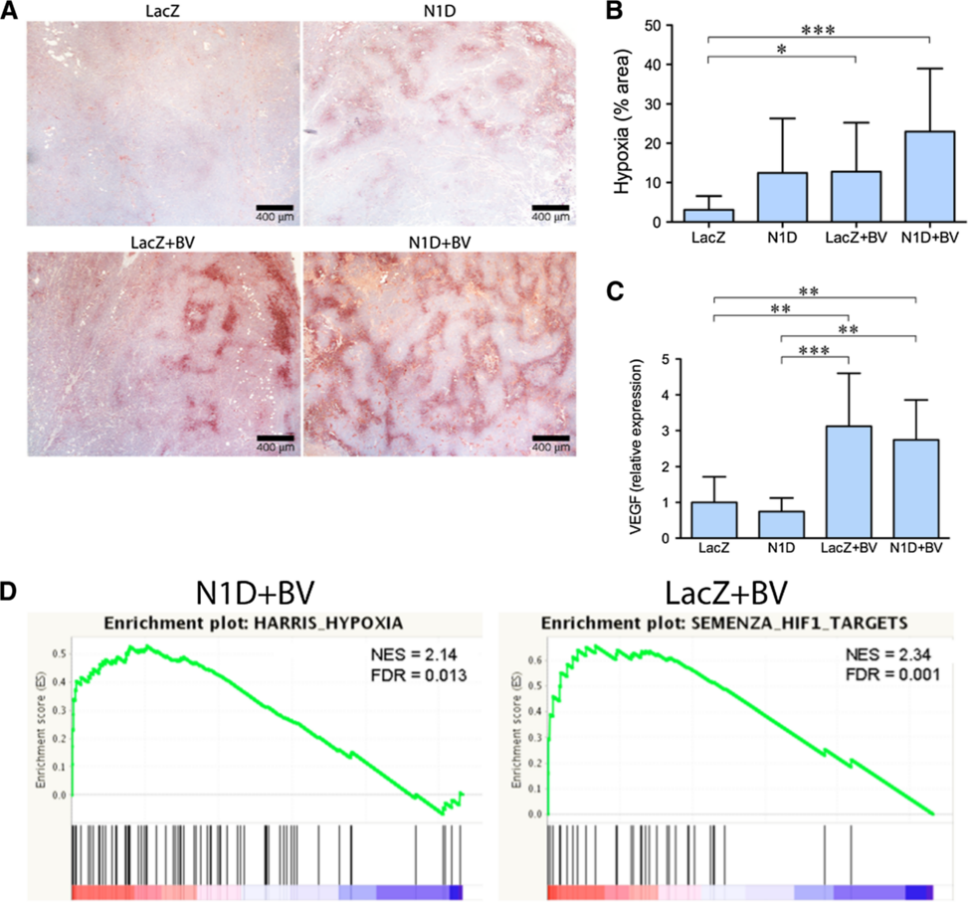
**Blockade of VEGF increases tumor hypoxia. (A)** Immunostaining for pimonidazole hypoxyprobe shows that NGP-LacZ controls (top left) show almost no detectable staining. Both NGP-N1D and NGP-LacZ + BV (top right and bottom left) show increased areas of Hypoxyprobe in some areas of the tumor, but wide areas remained well oxygenated. NGP-N1D + BV tumors (bottom right) show Hypoxyprobe staining throughout the tumor. Bar = 400 μm. **(B)** Quantification of Hypoxyprobe staining showed increased the hypoxia of NGP-LacZ + BV and NGP-N1D + BV compared to NGP-LacZ controls. Error bars represent SD. *P < 0.05, **P < 0.001. **(C)***VEGF* expression was increased in NGP-LacZ + BV and NGP-N1D + BV compared to NGP-LacZ or NGP-N1D. Error bars represent SD. **P < 0.01, ***P < 0.001. **(D)** GSEA enrichment plots are shown for HARRIS_HYPOXIA gene set NGP-N1D + BV vs NGP-LacZ, and for SEMENZA_HIF1_TARGETS NGP-LacZ + BV vs NGP-LacZ. Normalized Enrichment Score (NES) and False discovery rated (FDR) q-values are shown.

The lectin perfusion results demonstrate that combined Notch and VEGF blockade reduces both neoangiogenesis and VEGF blockade-induced vascular cooption. To determine if this resulted in increased hypoxia, we quantified pimonidazole (Hypoxyprobe®) by IHC. There was significantly more hypoxia in NGP-LacZ + BV (P < 0.05) and NGP-N1D + BV (P < 0.001) as compared to NGP-LacZ. (Figure 3A,B). NGP-N1D was not significantly different from NGP-LacZ tumors.

We also quantified the expression of two hypoxia responsive genes, *VEGF* and *PlGF*, in the tumors by real-time PCR. *VEGF* expression was significantly increased 3.1 fold (P < 0.01) in NGP-LacZ + BV and by 2.7 fold (P < 0.01) in NGP-N1D + BV tumors compared to NGP-LacZ controls and to NGP-N1D (Figure 3C). *PlGF* expression behaved in a similar pattern: relative to NGP-LacZ controls, NGP-N1D tumors had no change, while NGP-LacZ + BV tumors had 4.7 fold more (p < 0.001), and NGP-N1D + BV tumors had 3.79 fold more *PlGF* than controls (p < 0.001).

We further analyzed the tumors by gene expression profiling using HG-U133A 2.0 (human genome) Gene Chips (Affymetrix). *VEGF* expression was increased 2.36 fold in NGP-LacZ + BV and 2.56 fold in NGP-N1D + BV tumors (see Additional file 3 for significantly increased genes). Gene Set Enrichment Analysis (GSEA) [6] demonstrated that VEGF blockade resulted in enrichment of hypoxia related gene sets in comparison to control NGP-LacZ tumors (Table 1, Figure 3D). For NGP-N1D + BV there were 7 hypoxia related gene sets that were enriched (3 positively, 4 negatively); for NGP-LacZ + BV there were 3 hypoxia related gene sets that were enriched (3 positively); but none for NGP-N1D. Comparing NGP-LacZ + BV to NGP-N1D + BV did not show any significantly enriched gene sets, suggesting that Notch blockade did not further increase the hypoxia caused by VEGF blockade.

**Table 1:** Hypoxia associated gene sets* are enriched in BV treated tumors compared with LacZ tumors

**ENRICHED GENE SETS FOR N1D+BV TUMORS**				**ENRICHED GENE SETS FOR LacZ+BV TUMORS**			
**POSITIVELY ENRICHED GENE SETS**	**ES**	**NES**	**FDR q**-**val**	**POSITIVELY ENRICHED GENE SETS**	**ES**	**NES**	**FDR q**-**val**
**NAME**				**NAME**			
NIKOLSKY_BREAST_CANCER_17Q21_Q25_AMPLICON	0.600	2.850	0.000	XU_HGF_TARGETS_REPRESSED_BY_AKT1_DN	0.570	2.420	0.001
XU_HGF_TARGETS_REPRESSED_BY_AKT1_DN	0.640	2.510	0.000	***SEMENZA_HIF1_TARGETS***	***0.660***	***2.340***	***0.001***
REACTOME_PHASE_1_FUNCTIONALIZATION	0.810	2.260	0.005	SEIDEN_ONCOGENESIS_BY_MET	0.530	2.310	0.001
***SEMENZA_HIF1_TARGETS***	***0.660***	***2.220***	***0.005***	KEGG_ANTIGEN_PROCESSING_AND_PRESENTATION	0.530	2.300	0.001
KEGG_ANTIGEN_PROCESSING_AND_PRESENTATION	0.540	2.220	0.005	***HARRIS_HYPOXIA***	***0.530***	***2.290***	***0.001***
CUI_TCF21_TARGETS_DN	0.680	2.170	0.009	PRAMOONJAGO_SOX4_TARGETS_UP	0.580	2.260	0.002
***HARRIS_HYPOXIA***	***0.530***	***2.140***	***0.013***	HELLER_SILENCED_BY_METHYLATION_DN	0.480	2.160	0.011
NIKOLSKY_BREAST_CANCER_17Q11_Q21_AMPLICON	0.550	2.130	0.012	CUI_TCF21_TARGETS_DN	0.660	2.140	0.013
KEGG_DRUG_METABOLISM_CYTOCHROME_P450	0.560	2.120	0.014	REACTOME_RNA_POLYMERASE_I_PROMOTER_OPENING	0.560	2.140	0.011
KEGG_GRAFT_VERSUS_HOST_DISEASE	0.600	2.090	0.018	LIU_COMMON_CANCER_GENES	0.560	2.100	0.019
DAZARD_RESPONSE_TO_UV_SCC_UP	0.510	2.070	0.026	KEGG_SYSTEMIC_LUPUS_ERYTHEMATOSUS	0.460	2.080	0.022
LANDIS_BREAST_CANCER_PROGRESSION_UP	0.590	2.060	0.028	***LEONARD_HYPOXIA***	***0.580***	***2.080***	***0.022***
CHANNEL_REGULATOR_ACTIVITY	0.660	2.030	0.038	NOJIMA_SFRP2_TARGETS_UP	0.610	2.070	0.023
REACTOME_SYNTHESIS_OF_BILE_ACIDS_AND_BILE_SALTS	0.840	2.030	0.036	SPIRA_SMOKERS_LUNG_CANCER_DN	0.680	2.040	0.033
CYCLIC_NUCLEOTIDE_METABOLIC_PROCESS	0.840	2.010	0.044	RUNNE_GENDER_EFFECT_UP	0.830	2.020	0.042
SCHURINGA_STAT5A_TARGETS_UP	0.900	2.010	0.042	KEGG_LINOLEIC_ACID_METABOLISM	0.590	2.010	0.046
LU_TUMOR_ENDOTHELIAL_MARKERS_UP	0.700	2.000	0.044	NEBEN_AML_WITH_FLT3_OR_NRAS_DN	0.740	2.000	0.046
EXTRACELLULAR_LIGAND_GATED_ION_CHANNEL_ACTIVITY	0.660	1.990	0.047	LU_TUMOR_ENDOTHELIAL_MARKERS_UP	0.650	2.000	0.044
SEIDEN_ONCOGENESIS_BY_MET	0.500	1.990	0.046	**NEGATIVELY ENRICHED GENE SETS**			
ELVIDGE_HIF1A_TARGETS_DN	***0.480***	***1.980***	***0.045***	NIKOLSKY_BREAST_CANCER_7P22_AMPLICON	-0.820	-2.220	0.001
**NEGATIVELY ENRICHED GENE SETS**				**ENRICHED GENE SETS FOR N1D TUMORS**			
***ELVIDGE_HIF1A_TARGETS_UP***	-***0.610***	-***2.180***	***0.005***	**POSITIVELY ENRICHED GENE SETS**			
VANHARANTA_UTERINE_FIBROID_UP	-0.650	-2.180	0.003	**NAME**	**ES**	**NES**	**FDR q**-**val**
***ELVIDGE_HYPOXIA_DN***	-***0.520***	-***2.090***	***0.016***	NIKOLSKY_BREAST_CANCER_17Q11_Q21_AMPLICON	0.640	2.290	0.002
DACOSTA_ERCC3_ALLELE_XPCS_VS_TTD_UP	-0.710	-2.050	0.026	XU_HGF_TARGETS_REPRESSED_BY_AKT1_DN	0.610	2.240	0.004
NIKOLSKY_BREAST_CANCER_7P15_AMPLICON	-0.860	-2.050	0.025	LASTOWSKA_NEUROBLASTOMA_COPY_NUMBER_UP	0.550	2.210	0.003
GUENTHER_GROWTH_SPHERICAL_VS_ADHERENT_UP	-0.760	-2.030	0.029	KEGG_ANTIGEN_PROCESSING_AND_PRESENTATION	0.560	2.140	0.009
***ELVIDGE_HIF1A_AND_HIF2A_TARGETS_UP***	-***0.610***	-***2.000***	***0.045***	CHANNEL_REGULATOR_ACTIVITY	0.720	2.080	0.020
***ELVIDGE_HYPOXIA_BY_DMOG_DN***	-­‒0.580	-­‒1.980	0.048	AUXILIARY_TRANSPORT_PROTEIN_ACTIVITY	0.680	2.030	0.036
				REACTOME_PHASE_1_FUNCTIONALIZATION	0.760	2.010	0.041
				**NEGATIVELY ENRICHED GENE SETS**			
				GUENTHER_GROWTH_SPHERICAL_VS_ADHERENT_UP	-0.850	-2.160	0.010
				MATTIOLI_MULTIPLE_MYELOMA_SUBGROUPS	-0.810	-2.070	0.040
				MCGARVEY_SILENCED_BY_METHYLATION_IN_COLON_CANCE	-0.680	-2.040	0.042
				SCHLESINGER_METHYLATED_DE_NOVO_IN_CANCER	-0.570	-2.020	0.041

We used gene set expression analysis (GSEA) to identify significantly enriched gene sets (FDR q-val <0.05) in N1D+BV, LacZ+BV, and N1D tumors, compared with LacZ tumors. *HYPOXIA RELATED GENE SETS: ELVIDGE_HIF1A_TARGETS_DN (M2513) Genes down-regulated in MCF7 cells (breast cancer) after knockdown of HIF1A by RNAi; ***ELVIDGE_HIF1A_TARGETS_UP*** (M17905) Genes up-regulated in MCF7 cells (breast cancer) after knockdown of HIF1A by RNAi; ***ELVIDGE_HIF1A_AND_HIF2A_TARGETS_UP*** (M7062) Genes up-regulated in MCF7 cells (breast cancer) after knockdown of both HIF1A and HIF2A by RNAi; ***ELVIDGE_HYPOXIA_DN*** (M10175) Genes down-regulated in MCF7 cells (breast cancer) under hypoxia conditions; ***ELVIDGE_HYPOXIA_BY_DMOG_DN*** (M7030) Genes down-regulated in MCF7 cells (breast cancer) treated with hypoxia mimetic DMOG; ***HARRIS_HYPOXIA*** (M10508) Genes known to be induced by hypoxia; ***LEONARD_HYPOXIA*** (M19622) Genes up-regulated in HK-2 cells kidney tubular epithelium under hypoxia and downregulated on re-oxygenation; ***SEMENZA_HIF1_TARGETS*** (M12299) Genes that are transcriptionally regulated by HIF1A.

### Combined notch and VEGF blockade disrupts tumor vasculature

**Figure 4:**
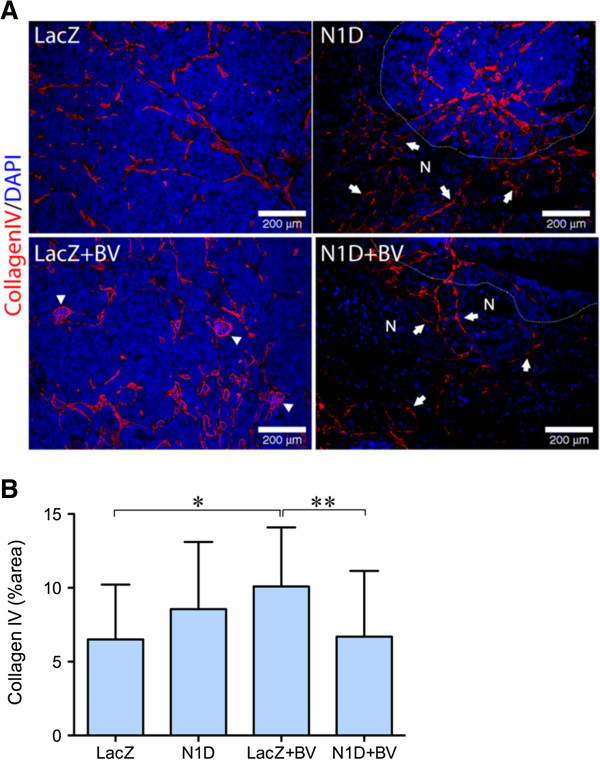
**Combined Notch and VEGF blockaded disrupts tumor vasculature. (A)** Immunofluorescence staining for Collagen IV (red) demonstrated vasculature (arrows) in areas of necrosis (N) as indicated by absence of nuclei by DAPI (blue), for NGP-N1D and NGP-N1D + BV. For clarity, a white dotted line delineates border between viable and necrotic tumor areas. In NGP-LacZ + BV arrowheads indicate coopted glomeruli. Bar = 200 μm. **(B)** Collagen IV was quantified, with NGP-LacZ + BV significantly higher in comparison to NGP-LacZ and NGP-N1D + BV. Error bars represent SD. *P < 0.05, **P < 0.01.

Combined blockade of VEGF and Notch reduced tumor viability, but did not further increase tumor hypoxia. To further understand the effect of combined blockade, we quantified tumor vasculature, using IHC for the basement membrane marker collagen IV, which forms a perivascular envelope that can persist after vascular regression. We detected a ~50% increase in collagen IV in NGP-LacZ + BV tumors as compared to both control NGP-LacZ and NGP-N1D + BV (Figure 4B), which suggested increased collagen IV contributed by coopted vasculature (Figure 4A, arrowheads). Notably, however, we noted persistent collagen IV in necrotic areas for NGP-N1D and for NGP-N1D + BV tumors (Figure 4A, areas denoted by “N” and delimited by dashed line). These results suggest that Notch blockade disrupts vasculature, with vascular regression marked by the collagen IV sleeves at sites of previously functioning vessels [7].

### Notch and VEGF blockade additively decrease survival of tumor endothelial cells

**Figure 5:**
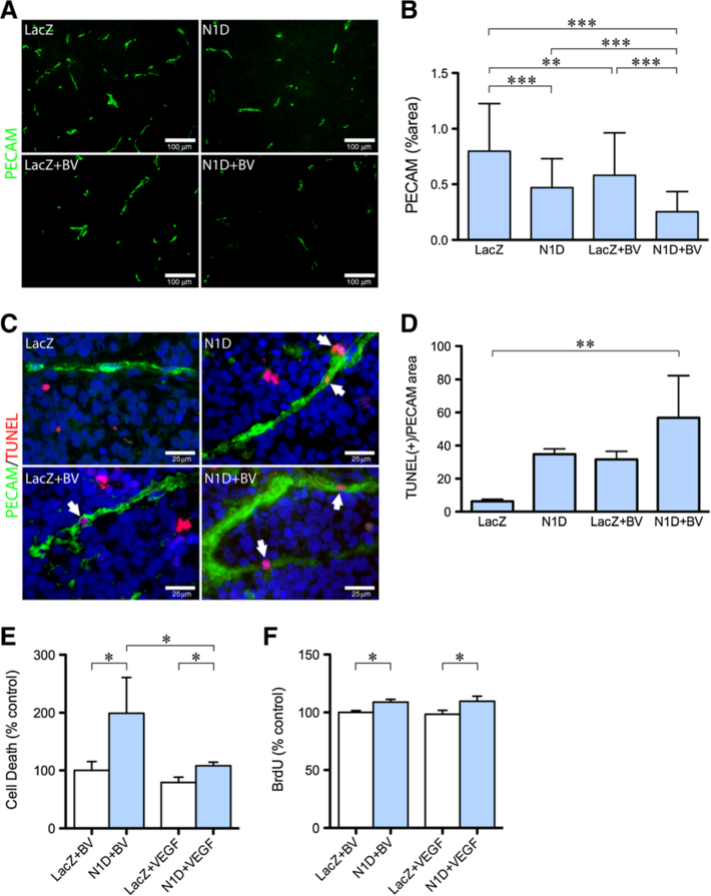
**Combined Notch and VEGF decreases endothelial cell coverage. (A)** Representative pictures of PECAM-1 immunofluorescence (green) of NGP-LacZ controls (upper left), NGP-N1D (upper right), NGP-LacZ + BV (lower left), and NGP-N1D + BV (lower right). Bar = 100 μm. **(B)** Quantification of PECAM-1 immunofluorescence. NGP-N1D and NGP-LacZ + BV tumors had decreased PECAM-1 than NGP-LacZ controls. NGP-N1D + BV tumors had an additive decrease in PECAM-1. Error bars represent SD. **P < 0.01, ***P < 0.001. **(C)** Representative pictures of triple staining for TUNEL (red), PECAM-1 (green), DAPI (blue), white arrowheads. Bar = 25 μm **(D)** The number of DAPI(+), TUNEL(+) cells surrounded by PECAM-1 in each tumor normalized by the viable area shows increased apoptotic ECs in NGP-N1D + BV tumors. Error bars represent SD. **P < 0.01. **(E)** HUVECs incubated with N1D and BV had increased apoptosis compared to HUVECs with BV only. HUVECs incubated with N1D and VEGF had decreased apoptosis compared to N1D with BV, but were not different from HUVEC incubated with BV only. Consistent with the known role of VEGF promoting endothelial cell survival, HUVEC incubated with VEGF had lower apoptotic levels than HUVECs incubated with BV, and lower apoptotic levels than HUVECs incubated with N1D and VEGF. Error bars represent SD. *P < 0.05. **(F)** N1D with BV resulted in increased BrdU incorporation by HUVECs compared to BV only. Addition of VEGF did not affect N1D-induced proliferation. Error bars represent SD. *P < 0.05.

We assessed endothelial cells in vasculature by IHC for PECAM-1 (Figure 5A). Combined VEGF and Notch blockade significantly decreased PECAM-1 staining (Figure 5B), compared to NGP-LacZ (68%), NGP-N1D (46%), and NGP-LacZ + BV (56%). These results along with the decrease in branching seen in the lectin perfusion studies, suggest that combined inhibition with NGP-N1D + BV decreases endothelial sprouting and neoangiogenesis.

Previous studies have suggested that both Notch and VEGF can act as survival signals for ECs. We therefore hypothesized that combined blockade would result in increased EC apoptosis. Quantification of apoptotic ECs was done using TUNEL (Figure 5C, red), PECAM-1 (Figure 5C, green), and nuclear DAPI immunostaining (Figure 5C, blue). A cell was considered an apoptotic EC when its nucleus, stained by DAPI, overlapped with TUNEL stain (red), and was surrounded by PECAM-1 (green) (Figure 5C, arrowheads). The number of apoptotic ECs in each section was then normalized to the PECAM positive area in each tumor. This quantification showed a significant increase in apoptotic EC in NGP-N1D + BV tumors (Figure 5D, P < 0.01). This increase in apoptotic ECs suggested that Notch and VEGF are both survival signals in tumor ECs, which act independently.

In order to test the effects of VEGF and Notch on EC survival *in vitro*, we used human umbilical vein endothelial cells (HUVEC). Conditioned media (CM) from NGP-N1D cells was collected as a source of N1D, with CM from NGP-LacZ as a control. CM from NGP-LacZ and NGP-N1D contained similar amounts of VEGF (1.26 ± 0.79 *vs.* 1.28 ± 1.4 ng/ml, respectively, P = n.s.). We therefore added 50 ng/ml of BV to block the VEGF that was present in the CM. We incubated the HUVECs in CM + BV or CM + VEGF (20 ng/ml of rhVEGF165) for 23 hrs and quantified apoptosis using a cell death ELISA. HUVECs incubated with N1D CM + BV had 1.5 to 2 fold higher apoptosis levels than HUVECs incubated with control CM + BV (Figure 5E). HUVEC apoptosis was significantly reduced when VEGF was added to the CM containing N1D (Figure 5E).

Inhibition of Notch signaling with γ-secretase inhibitors or anti-Dll4 antibodies have found that there is increased EC proliferation *in vitro*[8], while Notch1 activation leads to endothelial cell cycle arrest [9]. Using conditioned medium from NGP-LacZ or NGP-N1D cells, with either 50 ng/ml BV or 20 ng/ml rhVEGF165, we found that N1D caused a small increase (9%) in HUVEC proliferation *in vitro*, and which was not affected by addition of VEGF (10%, Figure 5F). Together, these data suggest that both endothelial proliferation and apoptosis increase simultaneously in NGP-N1D, as compared to NGP-LacZ tumors. ECs in NGP-N1D tumors treated with BV, proliferate more than controls, but also display more EC apoptosis than either N1D or BV treatment alone.

### Notch1 decoy and BV alter pericyte coverage of blood vessels

**Figure 6:**
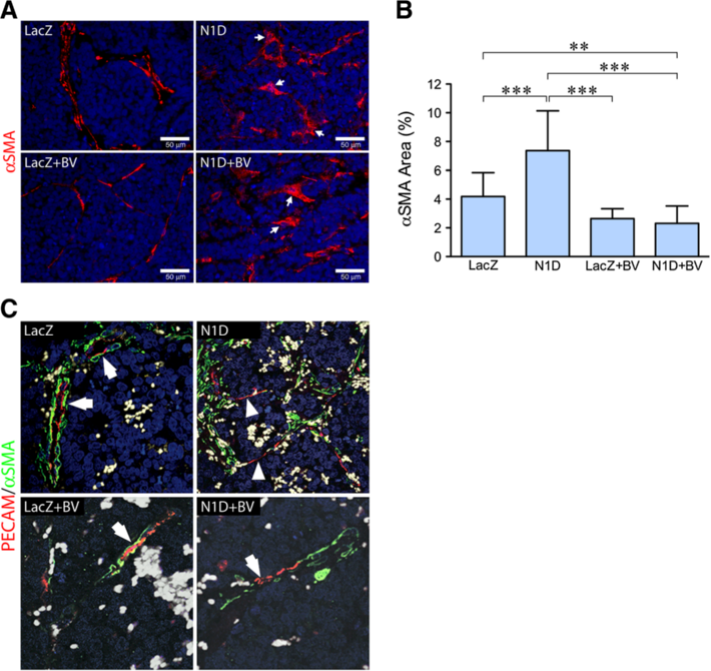
**Notch blockade disrupts pericyte interaction with endothelial cells. (A)** Immunofluorescence for the mature pericyte marker αSMA (red). NGP-N1D pericytes are discontinuous and their morphology is altered (right top, white arrows), compared to controls (left top). NGP-LacZ + BV vasculature had continuous coverage of pericytes. Pericytes of NGP-N1D + BV tumors showed discontinuity and altered morphology (bottom right, arrows). RBCs autofluoresce green. Bar = 50 μm. **(B)** Quantification of αSMA showed significant increase in NGP-N1D tumors, with a decrease in NGP-LacZ + BV and NGP-N1D + BV. Error bars represent SD. **P < 0.01, ***P < 0.001. **(C)** Confocal analysis of immunostaining for PECAM-1 (red) and αSMA (green). NGP-LacZ control tumors demonstrated PECAM-1(+) cells surrounded by αSMA(+) pericytes (upper left, arrows), NGP-Notch1 decoy tumors had stretches of PECAM-1(+) cells without surrounding αSMA(+) pericytes (upper right, arrowheads). NGP-LacZ + BV tumors had PECAM-1(+) cells surrounded by thin layers of αSMA(+) pericytes (lower left, arrow), while NGP-N1D + BV tumors had PECAM-1(+) cells not surrounded by αSMA(+) pericytes (lower right, arrowhead). RBCs are pseudocolored grey.

Immunostaining for the mature pericyte marker αSMA demonstrated discontinuity in this stromal cell envelope in NGP-N1D tumors as compared to NGP-LacZ, along with a more flattened morphology (Figure 6A, arrows), in agreement with our previous data [5]. BV treatment led to a thinner layer of pericytes, although immunostaining revealed a continuous pattern (Figure 6A, bottom left). Combined inhibition with N1D + BV, similar to N1D, led to discontinuity together with altered pericyte morphology (Figure 6A, right panels, white arrows). Quantification revealed an increase in total αSMA(+) area in NGP-N1D compared to the other groups (Figure 6B). NGP-N1D + BV had decreased αSMA(+) area compared to NGP-LacZ and NGP-N1D, indicating that combined blockade both diminishes the amount of pericytes coverage as well as disrupting the coverage (Figure 6B). Similar findings were seen using NG2, a marker of more immature pericytes (data not shown).

Confocal imaging for endothelial marker PECAM-1 and αSMA revealed that, while NGP-LacZ control tumor EC had neighboring pericytes (arrows), NGP-N1D tumors developed segments of ECs that lacked pericyte coverage (Figure 6C, right panel, arrowheads). NGP-LacZ + BV tumors contained ECs still covered by pericytes, although the pericyte layer was thinned compared to controls (Figure 6C, lower left panel). Similar to the NGP-N1D tumor vessels, NGP-N1D + BV vessels displayed segmental defects in pericyte coverage (Figure 6C, lower right, white arrows).

### Notch1 decoy effects on vasculature are direct

**Figure 7:**
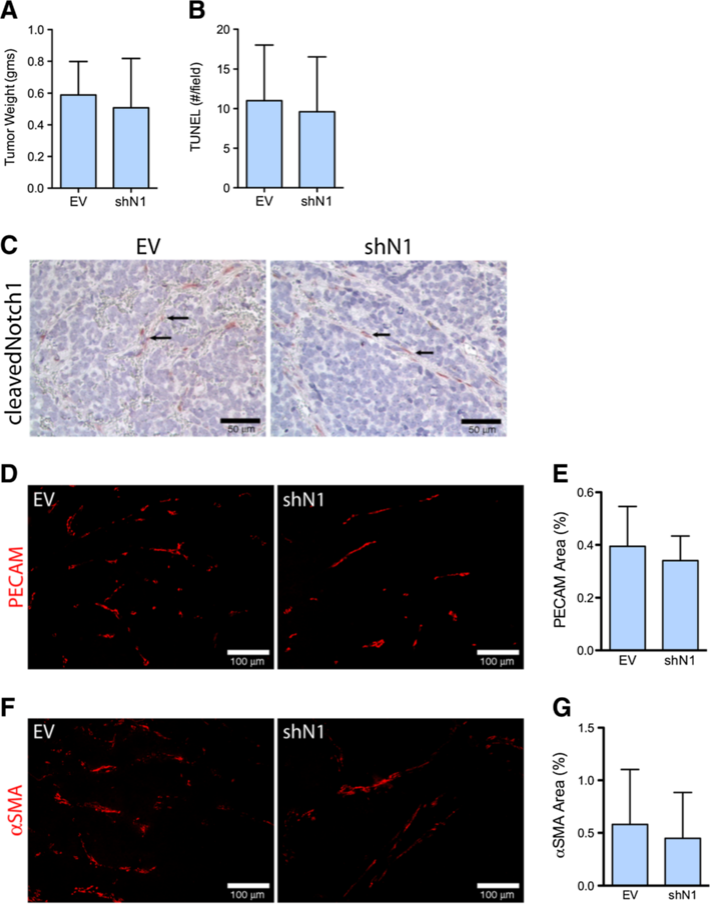
**Silencing of Notch1 does not recapitulate the vasculature seen with NGP-N1D. (A)** NGP-EV and NGP-shN1 tumor weights were not different. Error bars represent SD. **(B)** The number of TUNEL positive cells is not different between the NGP-EV and NGP-shN1 tumors. **(C)** Cleaved Notch1 is present in vasculature of NGP-EV and NGP-shN1 (arrows). **(D)** The EC marker PECAM-1 does not show discontinuity or lack of coverage in vasculature of NGP-shN1 tumors. Bar = 100 μm. **(E)** The percentage of PECAM-1 coverage did not differ between NGP-EV and NGP-shN1 tumors. Error bars represent SD. P = n.s. **(F)** The pericyte marker αSMA does not show erratic, discontinuous immunostaining in NGP-shN1 tumors. Bar = 100 μm. **(G)** There was no difference in percentage of αSMA coverage between NGP-EV and NGP-shN1 tumors. Error bars represent SD. P = n.s.

Immunofluorescence for cleaved Notch1 demonstrated Notch activation in vasculature of NGP-LacZ and NGP-LacZ + BV, but a paucity of activation in NGP-N1D and NGP-N1D + BV tumor vessels (Additional file 3). These data suggest that Notch blockade predominantly affects vascular cells. However, quantitative real time PCR analysis revealed that NGP tumor cells also express Notch1. In order to test the specificity of the effects of Notch blockade, we silenced Notch1 (shN1) in NGP. *In vitro,* NGP-shN1 had a 70% decrease in *Hes1* and 50% decrease in *Hey1* mRNA compared to empty vector (NGP-EV). *In vitro*, NGP-EV and NGP-shN1 cells proliferated at comparable rates, assessed by MTT assay (P = n.s.). We implanted the NGP-EV and NGP-shN1 cells into athymic mice and analyzed the tumors 6 weeks after implantation. We confirmed a 78 ± 18% (P < 0.05) reduction of *Notch1* mRNA, as well as reduction in expression of the Notch downstream target gene *Hes1* by 90% (P = 0.04)*,* in NGP-shNotch1 as compared to NGP-EV. We found no changes in tumor weight (Figure 7A), or apoptosis (Figure 7B) as a result of silencing Notch1. Immunostaining revealed that shN1 did not affect Notch1 activation in the tumor blood vessels (Figure 7C). The vasculature as assessed by percentage of PECAM-1 and αSMA positive area IHC, was similar in NGP-shNotch1 tumors compared to control NGP-EV tumors (Figure 7D-G). Lastly, the vasculature in NGP-shN1 tumors did not appear to be disrupted (Figure 7E,F), as was seen with Notch blockade (Figure 6A). Together, these data support the notion that the effects of N1D are due to Notch blockade in the tumor vasculature, rather than to an effect on the tumor cells.

## Discussion

A growing body of literature demonstrates crosstalk between Notch and VEGF pathways in angiogenesis *in vitro*[10-13], as well as in the mouse retina model [5,8,14,15]. The interaction between Notch and VEGF in tumor angiogenesis, however, is still in the process of definition. Our data suggests that Notch and VEGF have independent but coordinated functions in tumor angiogenesis, leading to additive effects when both pathways are simultaneously inhibited. VEGF blockade limits initial vessel sprouting and branching, and then later promotes vascular cooption [2] and the recruitment of pericytes that can stabilize the vasculature in the absence of VEGF [16,17]. Our results demonstrate that Notch blockade inhibits the later processes of cooption and EC/pericyte interaction.

Our data indicate that while VEGF blockade induces vascular cooption, this does not rescue tumors from hypoxia. Furthermore, while Notch blockade decreases cooption, this does not increase the VEGF-blockade induced hypoxia. This suggests that vascular cooption is relatively inefficient as a mechanism of providing oxygen to tumors; other potential contributions of co-opted vessels to tumor homeostasis remain unclear.

Our current results demonstrate that while Notch blockade can disrupt EC/pericyte interaction, it is only in the absence of VEGF that there is marked loss of vasculature, perfusion, and subsequent induction of tumor necrosis. In the context of loss of VEGF, Notch blockade causes EC apoptosis both *in vitro* and *in vivo.* This suggests that VEGF can rescue ECs when Notch is blocked.

Other groups have shown that antibodies against Dll4 and Notch1 increase non-functional EC vasculature in subcutaneous tumor models [8,14,15]. Endothelial Jagged1, however, has been shown to decrease sprouting in the mouse retinal model [18]. Therefore, the ability of the N1D to block both signaling by both Dll4 and Jagged1 [5] may explain the observed decrease in EC in NGP-N1D tumors. Our studies also suggest that the effect of Notch blockade on tumor angiogenesis is context-dependent. Thus, this effect may vary with different tumor models, and perhaps with tumor types.

## Conclusions

Our data lead us to propose that the Notch and VEGF pathways play distinct but complementary roles in tumor angiogenesis, and that Notch is required for VEGF-mediated vascular remodeling. Here we show that concurrent blockade disrupts primary tumor vasculature and viability further than inhibition of either pathway alone, and that manipulation of each pathway is reflected in distinct vascular defects. Further investigation of the interaction between VEGF and Notch signaling in vasculature may allow refinement of a combined approach to targeting tumor angiogenesis.

## Competing interests

The authors declare that they have no competing interests.

## Authors’ contributions

SLH contributed to design, and planned and executed mouse experiments, tissue analyses. DB, AG, AKC, and TK performed mouse tumor modeling, immunohistochemistry, and tissue analyses. WC, DA analyzed raw microarray data. YF, CJS, and JKK contributed to the design of the study and provided novel reagents. SLH, DB, DJY and JJK conceived of the study, supervised its execution, and wrote the manuscript. All authors read and approved the final manuscript.

## Supplementary Material

Additional file 1: Figure S1 N1D is expressed in NGP-N1D and NGP-N1D + BV tumors. **A)** Immunostaining for human Fc indicates the presence of N1D in NGP-N1D tumors (right panel, red), but not in NGP-LacZ tumors (left panel)**.** Bar = 200 μm **B)** SDS-Page shows the presence of N1D (upper band) in NGP-N1D + BV, but not in NGPLacZ + BV tumors. Presence of BV, which also contains Fc, is observed in both NGP-LacZ + BV and NGP-N1D + BV tumors (lower band). Click here for file

Additional file 2: Figure S2 Cleaved Notch1 activity is decreased in N1D tumors. Immunostaining for cleaved Notch1 (red) demonstrates increased Notch1 activity in vasculature of NGP-LacZ and NGP-LacZ + BV tumors, but nearly absent Notch1 activity in NGP-N1D and NGP-N1D + BV tumors. For NGP-LacZ + BV, cleaved Notch1 is seen in vascular cells surrounding a coopted glomeruli (green fluorescence due to fluorescein-labeled lectin). RBCs autofluoresce green/yellow. Nuclei are stained with DAPI (blue). Bar = 50 μm. Click here for file

Additional file 3 Genes up/down regulated in NGP-N1D+BV, NGP-N1D, NGP-LacZ+BV tumors. Click here for file
