# Supplementary material for: Notch and VEGF pathways play distinct but complementary roles in tumor angiogenesis
Source: Vasc Cell. 2013 Sep 25;5:17. doi: 10.1186/2045-824X-5-17 (PMC3849070; doi:10.1186/2045-824X-5-17)

Additional File 1. Figure S1. N1D is expressed in NGP-N1D and NGP-N1D+BV tumors.

A) Immunostaining for human Fc indicates the presence of N1D in NGP-N1D tumors (right panel, red), but not in NGP-LacZ tumors (left panel)**.** Bar=200 μm

B) SDS-Page shows the presence of N1D (upper band) in NGP-N1D+BV, but not in NGP-LacZ+BV tumors. Presence of BV, which also contains Fc, is observed in both NGP-LacZ+BV and NGP-N1D+BV tumors (lower band).

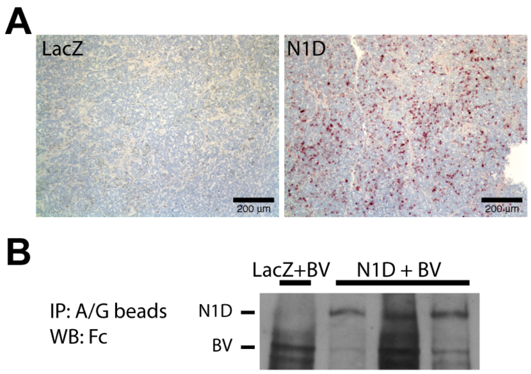

Supplement: Additional file 1: Figure S1 — N1D is expressed in NGP-N1D and NGP-N1D + BV tumors. A) Immunostaining for human Fc indicates the presence of N1D in NGP-N1D tumors (right panel, red), but not in NGP-LacZ tumors (left panel). Bar = 200 μm B) SDS-Page shows the presence of N1D (upper band) in NGP-N1D + BV, but not in NGPLacZ + BV tumors. Presence of BV, which also contains Fc, is observed in both NGP-LacZ + BV and NGP-N1D + BV tumors (lower band). [file 2045-824X-5-17-S1.pdf]
