# Supplementary material for: Notch and VEGF pathways play distinct but complementary roles in tumor angiogenesis
Source: Vasc Cell. 2013 Sep 25;5:17. doi: 10.1186/2045-824X-5-17 (PMC3849070; doi:10.1186/2045-824X-5-17)

Additional File 2. Figure S2. Cleaved Notch1 activity is decreased in N1D tumors

Immunostaining for cleaved Notch1 (red) demonstrates increased Notch1 activity in vasculature of NGP-LacZ and NGP-LacZ+BV tumors, but nearly absent Notch1 activity in NGP-N1D and NGP-N1D+BV tumors. For NGP-LacZ+BV, cleaved Notch1 is seen in vascular cells surrounding a coopted glomeruli (green fluorescence due to fluorescein-labeled lectin). RBCs autofluoresce green/yellow. Nuclei are stained with DAPI (blue). Bar=50 μm

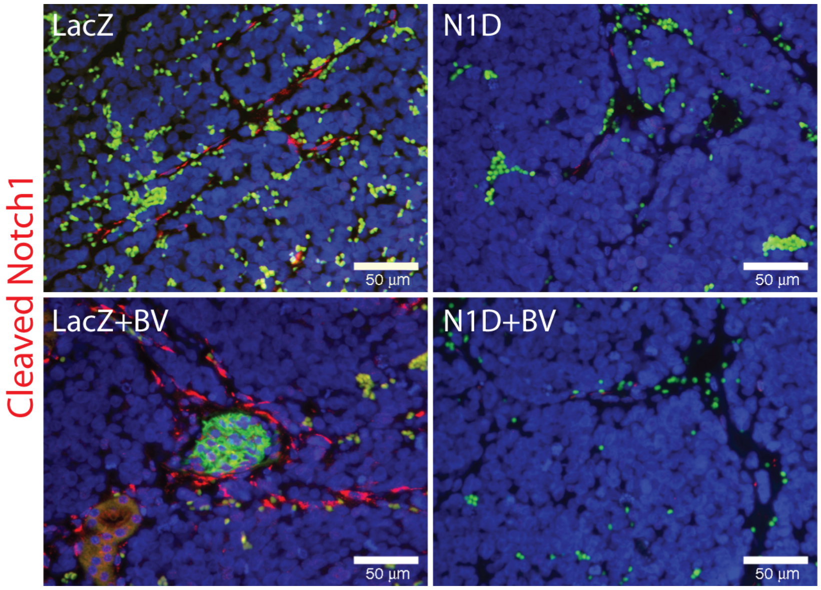

Supplement: Additional file 2: Figure S2 — Cleaved Notch1 activity is decreased in N1D tumors. Immunostaining for cleaved Notch1 (red) demonstrates increased Notch1 activity in vasculature of NGP-LacZ and NGP-LacZ + BV tumors, but nearly absent Notch1 activity in NGP-N1D and NGP-N1D + BV tumors. For NGP-LacZ + BV, cleaved Notch1 is seen in vascular cells surrounding a coopted glomeruli (green fluorescence due to fluorescein-labeled lectin). RBCs autofluoresce green/yellow. Nuclei are stained with DAPI (blue). Bar = 50 μm. [file 2045-824X-5-17-S2.pdf]
